# An existential perspective on physician-assisted dying in contemporary legislative debates

**DOI:** 10.1017/S1478951525100606

**Published:** 2025-09-12

**Authors:** Artin A. Mahdanian

**Affiliations:** 1 Independent Scholar, Washington, DC, USA; 2 Dr Artin Psychiatry, Washington, DC, USA; 3 Wellness Hub, McGill University, Montreal, QC, Canada

Ongoing conversations around Medical Assistance in Dying (MAiD) increasingly reflect the importance of grounding policy and practice in deeper moral and philosophical reflections, particularly as legislative and clinical contexts attract more attention (Attia et al. 2025). Recent parliamentary debates in several jurisdictions – including the UK, France, Scotland, Ireland, Germany, and some US states (notably Maryland and New York) – signal renewed legislative momentum on MAiD. In June 2025, the UK’s House of Commons passed the Terminally Ill Adults (End of Life) Bill, permitting terminally ill adults (prognosis < 6 months) to self-administer lethal medication with approval from 2 doctors and an expert panel. Meanwhile, France’s National Assembly approved a first-reading MAiD bill in late May, and Scotland’s Holyrood has begun a detailed review of its own proposal. As these legislative efforts advance, MAiD remains a highly controversial issue, generating passionate and principled arguments on both sides of the debate (Preston and Ost 2025).

Advocates ground their arguments in autonomy, compassion, dignity, and freedom, emphasizing individual suffering and personal choice at life’s end. They argue that MAiD complements high-quality palliative care for patients with refractory symptoms, and note that jurisdictions such as Canada, the Netherlands, Belgium, and Switzerland have implemented MAiD safely under strict criteria, often with high public approval (Frolic and Oliphant 2022). Critics, by contrast, raise concerns about coercion, inadequate safeguards, and evolving eligibility criteria. They caution that vulnerable individuals may feel pressured to choose MAiD for fear of being a burden, while disability and faith groups warn that safeguards – such as replacing judicial oversight with a panel review – may prove insufficient. Comparable debates in Belgium, the Netherlands, and Canada over mental health conditions and so-called “completed life” requests further underscore concerns about potential slippery slopes (Trimble 2025).

From a human rights standpoint, MAiD engages core principles: the right to life, dignity, autonomy, and freedom of conscience. Supporters argue that respect for individual liberty includes the right to choose the manner and timing of one’s death when faced with unbearable suffering. Existentialist perspectives similarly affirm the primacy of self-determination in confronting mortality, viewing such choices as deeply personal acts of freedom. Yet others contend that legal MAiD may alter societal obligations, shifting focus away from investment in palliative care, psychosocial support, and equitable health resources. These concerns highlight the need to balance personal rights with collective responsibilities (Mahdanian et al. 2023).

MAiD legislation raises complex medical, ethical, legal, cultural, religious, philosophical, and existential questions – such as autonomy versus protection, and personal dignity versus societal safeguards. As multiple legislatures move toward MAiD, policy design must remain sensitive to these tensions. Crucially, robust clinical infrastructure – encompassing palliative care, mental capacity, psychiatric evaluation, and professional training – must accompany legal frameworks, regardless of jurisdictional stance. Ensuring both respect for individual self-determination and protection from undue influence requires rigorous oversight, clear eligibility criteria, and sustained investment in end-of-life care systems.

The author confirms being the sole author of this Letter and takes full responsibility for its content. The views expressed are the author’s own and do not represent any organization. The author declares no competing interests, and no specific funding was received for this work.

## References

[ref1] Attia J, Kelly B and Best M (2025) Moral and philosophical frameworks for discussing Voluntary Assisted Dying (VAD). *Palliative and Supportive Care* 23, e92. doi:10.1017/S1478951525000331. 40190066 PMC13166321

[ref2] Frolic A and Oliphant A (2022) Introducing Medical Assistance in Dying in Canada: Lessons on Pragmatic Ethics and the Implementation of a Morally Contested Practice. *HealthCare Ethics Committee Forum: An Interdisciplinary Journal on Hospitals’ Ethical and Legal Issues* 34(4), 307–319. doi:10.1007/s10730-022-09495-7. PMC9437383 36053402

[ref3] Mahdanian AA, Laporta M, Drew Bold N, et al. (2023) Human rights in mental healthcare; A review of current global situation. *International Review of Psychiatry* 35(2), 150–162. doi:10.1080/09540261.2022.2027348. 37105153

[ref4] Preston N and Ost S (2025) The assisted dying bill continues through UK parliament, with legal momentum but also lingering questions. *British Medical Journal (Clinical Research Edition)* 389, r1220. doi:10.1136/bmj.r1220. 40514091

[ref5] Trimble M (2025) First steps down the slippery slope?: An analysis of the slippery-slope argument and its application to the question of assisted suicide. *The Ulster Medical Journal* 94(1), 42–46. PMID: 40313997; PMCID: PMC12042852. 40313997 PMC12042852

